# Past Marks: A Case Report of Basal Cell Carcinoma Induced by Arsenic Exposure

**DOI:** 10.7759/cureus.90674

**Published:** 2025-08-21

**Authors:** Patrícia Carreira, Mariana Coutinho, Sandra Lopez, Rita Constantino, Sara Almeida

**Affiliations:** 1 Planalto Family Health Unit, Lezíria Local Health Unit, Santarém, PRT; 2 Almeida Garrett Family Health Unit, Lezíria Local Health Unit, Santarém, PRT

**Keywords:** arsenic, basal cell carcinoma, carcinogenesis, fowler's solution, immunotoxicity

## Abstract

Basal cell carcinoma (BCC) is one of the most common forms of skin cancer worldwide. Chronic exposure to arsenic, particularly in its inorganic form, has been identified as a potential etiological factor in its development. However, not all individuals exposed to arsenic will develop BCC, suggesting that genetic and environmental factors influence susceptibility.

Arsenic was historically used for medicinal purposes, most notably in Fowler’s solution, once prescribed for conditions such as psoriasis. Despite its past therapeutic applications, arsenic is now classified as a carcinogen due to its well-established toxicity.

The authors present a case of a 69-year-old patient with a history of prolonged use of Fowler’s solution for psoriasis, who subsequently developed recurrent BCC, requiring continuous surveillance and treatment, such as surgery, cryotherapy, and laser therapy. Given the significant personal history of long-term exposure to an arsenic-based solution, arsenic was identified as the most plausible contributing factor in the development of cutaneous neoplasms in this case.

This case highlights the importance of a detailed medical history, including drugs or other toxic agents that the patient may have used previously. In particular, patients with known exposure to arsenic, especially for periods exceeding six months, should undergo lifelong surveillance due to the increased risk of developing skin cancer and other arsenic-related health conditions.

## Introduction

This case report presents the clinical trajectory of a 69-year-old woman with recurrent basal cell carcinoma (BCC) lesions appearing in both sun-exposed and covered areas, most plausibly attributed to inadvertent chronic arsenic exposure via Fowler’s solution. This case underscores the need to recognize historical medicinal arsenic use as a relevant etiological factor when evaluating multifocal or atypically distributed BCC.

Skin cancers are commonly categorized into melanoma and non-melanoma skin cancers (NMSC), the latter including basal cell, squamous cell, and Merkel cell carcinoma. BCC is the most common skin neoplasm, accounting for approximately 75% of NMSC cases [1,2].

BCC arises from a combination of genetic predisposition, individual host factors, and environmental exposures [1,2]. Ultraviolet (UV) radiation-particularly from sunlight the most significant environmental risk factor [1]. However, chronic exposure to inorganic arsenic, whether through contaminated water, food, or medicinal preparations, has also been strongly implicated in its pathogenesis [1,2].

There are several histological subtypes of BCC, classified by their invasiveness: nodular, superficial, morpheaic, and basosquamous. The nodular subtype, comprising approximately 60% of BCCs, typically presents as a well-circumscribed mass with telangiectasia. Superficial lesions account for roughly 20% and appear as flat, erythematous patches with well-defined margins. Morpheaform lesions are described as scar-like, whitish plaques with ill-defined borders and may present with ulceration or pigmentation [1].

Most BCCs develop in sun-exposed areas, with up to 70% affecting the face, further emphasizing the role of UV radiation. When lesions occur in less sun-exposed areas, such as the inner thighs or genitals, other etiological factors should be considered [1]. BCC associated with arsenic exposure can develop on both sun-exposed and non-exposed skin sites [2]. 

For experienced clinicians, BCC is often suspected based on clinical examination and dermoscopic features. Dermoscopy may reveal characteristic features such as ovoid nests, leaf-like structures, telangiectasias, erosions, pigmentation, or absence of a pigment network. Nevertheless, skin biopsy remains the gold standard for definitive diagnosis and histologic classification [1,3].

Surgical excision is the first-line treatment for most BCCs. However, additional therapies such as radiotherapy, cryotherapy, immune response modifiers (e.g., imiquimod), and hedgehog pathway inhibitors (e.g., vismodegib or sonidegib) have expanded treatment options [3]. 

Although treatment options for BCC have evolved, it remains crucial to understand the etiological factors, especially those linked to environmental or iatrogenic exposure, as these may be preventable. One such factor is arsenic, a well-documented carcinogen that has historically been used in medical treatments. In particular, Fowler´s solution - a potassium arsenite preparation - was widely prescribed for chronic conditions such as psoriasis [4-6]. 

Psoriasis is a chronic inflammatory condition characterized by erythematous plaques with thick, silvery scales. Despite the availability of diverse therapeutic options, psoriasis remains a chronic, incurable disease, and its relapsing course and impact on the quality of life have led, over the years, to the use of a wide range of treatments, some of which are now known to carry significant long-term risks, like Fowler’s solution. Current therapies aim to achieve long-term disease control, reduce symptoms, and improve patients' quality of life [4].

Although arsenic showed some efficacy in controlling psoriatic lesions, its toxicity is well-documented, and its medical use has since been discontinued. The World Health Organization (WHO) defines chronic arsenic toxicity as *arsenicosis*, characterized by skin changes such as hyperpigmentation, palmar-plantar hyperkeratosis, and an increased risk of malignancies (especially skin cancers), peripheral vascular disease, and systemic toxicity [5,7].

Arsenic exists in both organic and inorganic forms, with inorganic arsenic being significantly more toxic [5,7]. It can be absorbed through ingestion, inhalation, or dermal exposure [8]. Once in the body, arsenic interferes with biochemical processes by mimicking substances like phosphate, substituting in molecules such as adenosine triphosphate (ATP). The liver attempts detoxification through methylation; the key enzyme in this process is the arsenite methyltransferase (AS3MT), which converts arsenic into more excretable forms [9]. Inefficient methylation leads to arsenic accumulation in organs, resulting in long-term cellular and genomic damage that heightens cancer risk [8]. Due to its established carcinogenicity, arsenic is classified as a Group 1 carcinogen by the International Agency for Research on Cancer (IARC) [7].

Arsenic exposure has been associated with various skin malignancies, including BCC or squamous cell carcinoma [7,10]. However, not all exposed individuals develop cancer, suggesting that genetic and immunologic factors modulate individual susceptibility [1,7]. 

This case report aims to describe a patient with recurrent BCC likely related to past arsenic exposure, illustrate the diagnostic and therapeutic challenges associated with arsenic-induced BCC, and highlight the public health relevance of historical arsenic use, particularly in patients previously treated with arsenic-containing therapies. This report also emphasizes the importance of obtaining a comprehensive exposure history and the need for long-term dermatologic surveillance in individuals with known arsenic exposure.

## Case presentation

A 69-year-old woman, a retired schoolteacher, presented with a medical history significant for psoriasis, hypertension, dyslipidemia, depressive disorder, Sjögren’s syndrome, Hashimoto’s thyroiditis, autoimmune gastritis, and osteoporosis. She reported sun exposure limited to the summer months, during which she consistently used sunscreen. She denied any history of smoking or alcohol consumption, and there was no known family history of cancer.

The patient was diagnosed with psoriasis vulgaris at the age of 10. At age 17, she was prescribed Fowler’s solution for psoriasis management. Although the patient could not recall the exact dose or frequency, she reported using it for approximately 8 to 10 months, during which she experienced significant remission of psoriatic lesions. No medical records from that period were available to confirm these details, limiting the ability to quantify cumulative arsenic exposure and establish a direct dose-response relationship. Nonetheless, the prolonged use during adolescence likely contributed to cumulative arsenic toxicity. Approximately four years after treatment with Fowler’s solution, she experienced recurrent psoriatic flare-ups, which have since been effectively managed with topical therapy.

At age 34, she received her first diagnosis of BCC. Over the following years, she developed multiple recurrences, with lesions affecting both sun-exposed areas (face, scalp, and neckline) and less sun-exposed regions (dorsal trunk, lumbar area, inguinal region, and lower limbs). Given her documented history of arsenic exposure and the distribution of lesions, her dermatologist considered arsenic to be the most probable etiological factor underlying the recurrent BCCs.

Following her initial BCC diagnosis, she commenced regular dermatologic follow-up and underwent multiple procedures, including cryosurgery and laser therapy. At age 64, due to persistent recurrences, treatment with vismodegib was initiated. However, after six weeks, she developed toxic hepatitis that required hospitalization, and the medication was permanently discontinued. 

**Figure 1:**
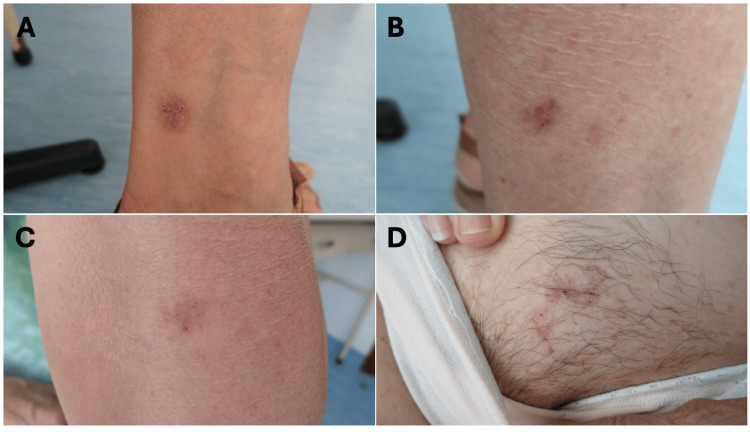
Basal cell carcinomas in the following locations: (A) lateral side of the right leg, (B) posterior side of the right leg, (C) posterior side of the right arm, and (D) pubic region.

Due to this complication, she discontinued follow-up care at the public hospital dermatology department and continued treatment with a private dermatologist. In June 2023, during a routine consultation with her family physician, she requested a referral back to the public dermatology department. At that visit, she presented four new cutaneous lesions with clinical features highly suggestive of BCC, all of which were subsequently excised surgically (Figure 1).

The patient has since become highly proficient at self-identifying suspicious skin lesions, allowing for early detection and timely removal. She currently has regular follow-ups with the hospital’s dermatology department for ongoing surveillance and prompt management of any recurrences.

## Discussion

Arsenic exposure is a global public health concern due to its widespread environmental presence and association with numerous adverse health effects. According to the WHO, more than 140 million people in over 70 countries are exposed to drinking water with arsenic concentrations exceeding the recommended limit of 10 µg/L [11]. Arsenicosis, defined by the WHO, is a chronic health condition resulting from prolonged exposure to arsenic - typically over at least six months at toxic doses [5,11]. The Centers for Disease Control and Prevention (CDC) defines toxic exposure as 0.005 mg/kg/day for acute and 0.0003 mg/kg/day for chronic exposure [12,13]. Arsenic contamination primarily occurs through ingestion of contaminated water and food; however, inhalation - particularly in industrial and agricultural settings - is also a recognized route [11].

Historically, arsenic has been used both as a potent toxic and a therapeutic agent. During the 18th to early 20th centuries, Fowler’s solution contained approximately 1% potassium arsenite (equivalent to approximately 7.6 mg elemental arsenic or 1 mg of arsenic trioxide per mL), and it was widely prescribed for conditions such as malaria, syphilis, asthma, leukemia, and chronic dermatological diseases like eczema and psoriasis [5]. At the time, arsenic was believed to regulate aberrant skin cell proliferation characteristic of psoriasis [6]. It was also observed that Fowler’s solution could reduce leukocyte count in patients with leukemia [5].

In early 20th-century clinical use, Fowler’s solution was typically prescribed in doses of 5 to 15 drops (≈0.25-0.75 mL) one to three times daily, sometimes for periods ranging from several months to years [13]. Such regimens could produce substantial cumulative arsenic exposure; for example, historical dosing patterns suggest that 8-10 months of use, as reported by our patient, could plausibly result in cumulative exposure in the gram range, a level consistent with long-term toxicity thresholds described in epidemiological studies [14]. 

Despite its observed benefits, the high toxicity and carcinogenic potential of arsenic led to the discontinuation of its therapeutic use in this form [5]. Nevertheless, arsenic trioxide remains in limited medical use today, particularly in hematology, where it is employed in the treatment of acute promyelocytic leukemia. In this context, it promotes blast cell differentiation and stimulates immune system activity, highlighting the complex dual nature of arsenic as both a therapeutic agent and a carcinogen [5,7].

Arsenic is currently considered by the IARC of the WHO as a class I carcinogen [7], and its toxicity is well known, especially in its inorganic form [11]. Arsenic exerts its toxicity through multiple mechanisms, including oxidative stress, DNA damage, inhibition of repair pathways, chromosomal abnormalities, epigenetic modifications, and dysregulated apoptosis. In cutaneous tissue, these changes result in abnormal keratinocyte proliferation, differentiation, dysplasia, and impaired immune regulation, all of which are implicated in carcinogenesis [7,10,15]. 

Although arsenic exposure is widespread, only approximately 1% of individuals develop skin cancer, suggesting that individual susceptibility - particularly genetic and immune-related factors - plays a significant role [7]. In particular, mutations in the AS3MT gene, encoding the enzyme AS3MT, which plays a fundamental role in arsenic detoxification, have been associated with increased risk of arsenic-induced cutaneous lesions [1,16].

A key molecular mechanism in BCC pathogenesis is dysregulation of the Sonic Hedgehog (Shh) signaling pathway. Under physiological conditions, the PTCH1 gene encodes a receptor that suppresses the activity of Smoothened (SMO), a key Shh pathway effector. When the Shh ligand binds PTCH1, this inhibition is lifted, enabling SMO to activate GLI transcription factors, which then upregulate genes involved in cellular proliferation. Mutations in PTCH1 - frequent in BCC - or arsenic-induced impairment of GLI3 repressor formation may result in constitutive activation of the pathway and unregulated cell proliferation [1-3]. 

This understanding has led to targeted therapies such as vismodegib, a Hedgehog pathway inhibitor that blocks SMO activity. Vismodegib is approved for the treatment of advanced or metastatic BCC, especially in cases where surgery or radiotherapy are contraindicated [3].

In our patient, toxic hepatitis developed six weeks after starting vismodegib. Although uncommon, vismodegib-associated drug-induced liver injury (DILI) is documented in clinical experience, post-marketing safety analyses, and case reports. Mechanistically, the injury appears idiosyncratic, with a suspected hypersensitivity component; vismodegib is hepatically metabolized via multiple CYP isoenzymes (CYP3A4, 2C8, 2C9, 2C19) and has a prolonged half-life (~19 days), which may increase vulnerability in predisposed hosts [17]. Regulatory labeling notes elevations in liver enzymes and hepatitis events [18], while published case reports describe cholestatic or mixed-pattern injury, typically arising within weeks to two months of therapy and improving after drug withdrawal [19,20]. In this case, additional susceptibility may have stemmed from age, polypharmacy, and autoimmune comorbidities (e.g., Sjögren’s and Hashimoto’s), which are recognized clinical risk contexts for idiosyncratic DILI.

Arsenic poisoning manifests in two clinical forms: acute and chronic, with chronic exposure being the most relevant in our case due to its established association with cutaneous carcinogenesis. Chronic exposure to arsenic leads to multi-organ arsenic accumulation, particularly in the skin, liver, lungs, heart, kidneys, and, to a lesser extent, in the muscles, nervous system, gastrointestinal tract, and spleen. [9] Among affected organs, the skin is particularly susceptible, with dermatological changes such as hyperpigmentation and hyperkeratosis often preceding malignant transformation into Bowen's disease, BCC, and squamous cell carcinoma. These lesions may appear decades after exposure [7,10].

Epidemiological studies strongly support the link between chronic arsenic exposure and non-melanoma skin cancers. A large population-based cohort study using data from the National Taiwan Cancer Registry (1979-2007) demonstrated a three- to fourfold increase in BCC incidence in areas endemic for blackfoot disease - a condition strongly associated with arsenic exposure - compared to non-endemic regions [14].

This report highlights the association between chronic arsenic exposure and the development of BCCs. Unlike radiation-induced BCCs, which typically occur in sun-exposed areas, the distribution of lesions in this patient aligns with the pattern seen in arsenic-related BCC, as these tumors frequently occur in non-sun-exposed sites. The latency period of approximately 17 years between exposure and the first BCC diagnosis is also consistent with previously reported data on arsenic-induced carcinogenesis [2,7]. Although the patient did not undergo molecular testing for mutations in genes such as PTCH1 or AS3MT, which have been associated with increased susceptibility to BCC and altered arsenic metabolism, and no biomarker measurements (e.g., hair, nails, urine, tissue) were performed to confirm residual body burden, the minimal lifetime UV exposure combined with the multifocal distributions of lesions remain strongly aligned with epidemiological and clinical descriptions of arsenic-induced BCC [2,7,14].

This case also underscores preventive opportunities that extend beyond individual awareness. At a population level, arsenic-related health risks call for structured public health measures, such as routine groundwater monitoring, arsenic-removal technologies, and education campaigns in communities with historical or ongoing exposure [11]. At the clinical level, the absence of risk-based screening likely delayed recognition of the patient’s susceptibility [14]. Individuals with a documented history of arsenic exposure-such as those treated with Fowler’s solution-should be classified as an at-risk group, warranting annual dermatologic examinations, a low threshold for biopsy of atypical lesions, and periodic laboratory monitoring of systemic function. Notably, this patient now performs effective self-monitoring, illustrating the value of patient education in facilitating earlier detection. Implementing such preventive strategies, combining population-level interventions with risk-based clinical surveillance, could help reduce the long-term impact of arsenic-related skin cancer.

## Conclusions

This case illustrates the long-term carcinogenic potential of arsenic, historically prescribed as Fowler’s solution, and its likely contribution to multiple recurrent BCCs in our patient. It underscores the importance of obtaining a comprehensive clinical history, with particular attention to past exposure to toxic agents. Patients with known arsenic exposure should undergo long-term dermatologic surveillance, ideally with annual evaluations, due to their increased risk of developing cutaneous malignancies. Arsenic remains a public health concern due to past medicinal use and ongoing environmental exposure, underscoring the need for broader public health strategies, including awareness and screening initiatives, to mitigate its associated disease burden.
